# Maternal and Congenital cytomegalovirus infection and zero rubella IgM prevalence in newborns in St.Paul’s Hospital Millennium Medical College

**DOI:** 10.1186/s13104-016-2274-1

**Published:** 2016-10-21

**Authors:** Yeshwondm Mamuye, Balkachew Nigatu, Delayehu Bekele, Mekonen Getahun

**Affiliations:** 1 Department of Microbiology, Immunology and Parasitology, St.Paul’s Hospital Millennium Medical College, P.O.Box 1271, Addis Ababa, Ethiopia; 2 Department of Obstetrics & Gynecology, St.Paul’s Hospital Millennium Medical College, P.O.Box 1271, Addis Ababa, Ethiopia; 3 Ethiopian Public Health Institute (EPHI), Addis Ababa, Ethiopia

**Keywords:** Congenital, Cytomegalovirus, Rubella, Ethiopia

## Abstract

**Background:**

Maternal cytomegalovirus (CMV) and Rubella infections result in adverse neonatal outcomes. Both CMV and Rubella are more widespread in developing countries and in communities with lower socioeconomic status. Thus, the aim of this study was to determine IgM specific to CMV and *Rubella* among newborns and Maternal CMV-seroprevalence and to identify risk factors.

**Method and finding:**

Using cross sectional study design a total of 312 (156 newborns and 156 mothers) study participants were recruited by simple random sampling technique from gynecology outpatient department (OPD) and ward, starting from April 1, 2015 to June 30, 2015. Cord and venous blood samples were collected from all participants and structured questionnaire was introduced to gather risk factor related data. ELISA was used to detect CMV and Rubella-IgM. SPSS version 20 was used to analyze the data, and regression analysis was also performed. Out of 156 newborns, 2 [1.3 %; 95 % CI: 0.0–3.8] were positive for CMV—IgM and no single rubella was detected. Association was not computed between risk related variables and cytomegalovirus infected newborns due to the low positivity rate. Multiple independent predictors were found between maternal CMV-IgM and Obstetrical characteristics. Cytomegalovirus—IgM was significantly isolated from mothers with history of transfusion (25.0 %, OR 0.09, 95 % CI 0.0–0.3, P = 0.006), history of abortion (OR 0.02, 95 % CI 0.0–0.6, P = 0.023), HIV sero-status (OR 5.0, 95 % CI 1.5–15.8, P = 0.034), and multi parity (OR 0.08, 95 % CI 0.01–0.7, P = 0.022).

**Conclusion:**

Although low congenital CMV and no Rubella are reported among newborns, more effort is needed to screen for congenital infectious viral disease as well as usage of advanced techniques should be taken into consideration.

**Electronic supplementary material:**

The online version of this article (doi:10.1186/s13104-016-2274-1) contains supplementary material, which is available to authorized users.

## Background

Human *cytomegalovirus* (CMV) is a member of the family Herpesviridae and belongs to the subfamily betaherpesviridae. CMV has worldwide distribution, infects humans of all ages and all socioeconomic groups, and with no seasonal or epidemic patterns of transmission [1]. It is the most common cause of con-genital infection with birth prevalence of about a range of 0.2–2.5 percent, and a common cause of deafness and intellectual impairment worldwide [2–4].

In *utero* transmission of CMV can occur following primary maternal infection during pregnancy but can also occur in women with natural immunity, either because of the reactivation of latent virus or by re-infection with a different strain [5]. Postnatally, CMV is also transmitted from mother to child through breastfeeding and close contact [6]. The transmission risk is the proportion of mothers undergoing a primary infection in a given trimester and/or the preconception period who transmitted CMV to the fetus [7].

Maternal infection especially during the first trimester associated with adverse neonatal outcome which encompass heart disease, cataract and deafness collectively known as congenital rubella syndrome which had a major neonatal morbidity and burden to families [8]. Although, incidence of rubella infection is reduced worldwide, some African countries like Mozambique still have a high incidence (95.3 %) [9, 10]. Rubella vaccine is cost-effective and cost-beneficial, therefore since year 2000 WHO proposed an introduction of rubella vaccine program in each country [11].

Cytomegalovirus is one of the most common causes of congenital infections; however, in Ethiopia CMV infection rate among newborn is yet undetermined and this might worsen the outcome of the disease.

The aim of the study was to determine IgM specific to CMV and Rubella in newborns who delivered at Department of Obstetrics and Gynecology of St. Paul’s Hospital Millennium Medical College. The recognition of congenital CMV and *Rubella* among newborns in the country, helps to develop effective prevention and treatment protocols. Therefore, this study adds an important input on CMV burden data to design appropriate control measures for Ethiopian mothers and children. This study also, highlights improving awareness to clinicians and creating a public concern for the communities.

## Methods

A cross sectional study was conducted at St. Paul’s Hospital Millennium Medical College (SPHMMC), Addis Ababa, Ethiopia. It is located in an urban setting and serves as both a tertiary hospital for Ethiopia and teaching hospital for national and international students. The hospital provides out and in patient services with a total of 372 beds. Accordingly, patients being seen at SPHMMC came from all over Ethiopia. The sample size for the study was calculated using the formula (n = (zα/2)2 p (1-p)/d2) for estimating a single population proportion at 95 % confidence interval (CI) (Zα/2 = 1.96), 5 % margin of error, and 10 % non-respondents rate based on IgM specific prevalence of CMV from a study in Sub-Saharan region among infants 3.8 % [12]. Then a total of 56 sample size was calculated. However, by considering non respondent rate and design effect, the total number of newborn were increased to156. Therefore, 156 mothers were recruited using simple random sampling technique.

### Data collection

Cord and venous blood samples were collected from selected study participants, who gave birth in gynecology and obstetrics clinic, starting from April 1, 2015 to June 30, 2015. Serum from cord samples were collected under aseptic conditions, and transported using ice-box to Ethiopian Public Health Institute (EPHI) and examined for the presence of CMV and *Rubella* specific IgM. Maternal serum samples were screened for CMV-IgG and CMV-IgM in St.Paul’s Hospital Millennium Medical College clinical chemistry department.

Standard structured questionnaires were designed to collect information regarding socio-demographics characteristics of the mother and risk related (weight, height, head circumference of the new born and maternal HIV sero-status, HBsAg, blood transfusion history,, history of abortion frequency of pregnancy and number of under five children) data. The questionnaire was first developed in English and translated into local language, Amharic. Questions were pre-tested in non-selected health institutions to assess the content validity, appropriateness, and question comprehensibility. The questionnaire was revised accordingly. Three data collectors from the institution in the study area were selected. Training was given to the data collectors for two day on how to conduct the interview, content of the questionnaire, data quality, and ways to approach respondents. The first author checked the questionnaires for completeness every day. Incomplete questionnaires were excluded. Five percent of the interviewed participants were randomly selected and re-interviewed by the first author.

### Laboratory method

Cord blood was screened for CMV-specific IgM using the ELISA test kits (Diagnostic Automation, Inc., USA) according to manufacturer’s guideline. Briefly, purified CMV antigen is coated on the surface of micro wells. Participant’s serum was then added to wells. If antibody is there in the serum, then it would bind to the antigen that is coated on the well. All unbound materials are washed away and an enzyme conjugate is added to the well. The conjugate, then binds to the antibody-antigen complex. Excess enzyme conjugate is washed off and TMB Chromogenic Substrate is added. Intensity of the color generated by the bound conjugate is proportional to the amount of IgM specific antibody present in the sample. Results are then read by a micro-well reader compared in a parallel manner with calibrator and controls. Quantitative analysis for CMV and *Rubella* specific IgM (Siemens, Germany) were performed, and the assay result interpreted as IU/mL. The manufacturer’s instructions were followed for the cutoff points, which was <1.1 IU/mL for CMV IgG and IgM. Results <1.0 OD value was considered negative for *Rubella* IgM. Whereas, maternal venous blood was analyzed by using Elecsys (cobas e 411) Roche reagent screened for CMV specific for IgG and IgM.

### Data analysis

The data were entered (with double entry) and cleaned with *Epidata version 3.1*, and analyzed using SPSS *version* 20. Statistical significance was considered when *P* value <0.05. newborn CMV specific IgM and maternal CMV-IgG & IgM prevalence was determined by dividing the number of infected individuals by the total number of individuals screened for CMV infection. Frequency distribution tables were used to quantify participant’s age range, gestation, occupation, parity and risk factors of CMV positivity rate. Newborns that had positive result for CMV specific IgM infection were low in number and therefore, a regression was not computed.

However, logistic regression analysis was used to quantify the effect of different clinical and obstetrical related risk factors on maternal CMV seroprevalence. 95 % confidence intervals were calculated for odds ratio. Values were considered statistically significant when P-value <0.05.

## Results

### Socio demographic characteristics

A total of 312 study participants (156 infants and 156 mothers) were enrolled. Eighty-one male and 75 female newborns with a mean and standard deviation of weight in gram, height, and circumference in cm, 3.0 ± 0.6, 41.9 ± 6.87, and 32.7 ± 3.1 respectively. The mean age of the mothers was 26.2 ± 5.0, with age range of 18-45. Of 156 study participants, 48.7 % were under 25. The majority of the participants (46.2 %) had at least secondary education. The majority were married (99.4 %) and 82.7 % were housewives (Table 1).

**Table 1:** Distributions of Cytomegalovirus infection along with maternal (n = 312) demographic characteristics, St.Paul’s Hospital Millennium Medical College, Addis Ababa, 2015

Characteristics		Seroprevalence		Total n (%)
		Positive n (%)	Negative n (%)	
Sex newborn	Male	NA	NA	81 (51.9)
	Female	NA	NA	75 (48.1)
Income ethiopian birr	+1500			81 (51.9)
	1500–2500			55 (35.3)
	>2500			20 (12.8)
Maternal age	+25	73 (49.0)	3 (42.9)	76 (48.7)
	26–35	69 (46.3)	4 (57.1)	73 (46.8)
	36+	7 (4.7)	0	7 (4.5)
Marital status	Married	148 (99.3)	7 (100.0)	155 (99.4)
	Others	1 (0.7)	0	1 (0.6)
Educational	Illiterate	32 (21.5)	2 (28.6)	34 (21.8)
	Primary	41 (27.5)	0	41 (26.3)
	Secondary	67 (45.0)	5 (71.4)	72 (46.2)
	Higher education	9 (6.0)	0	9 (5.8)
Occupation	Housewife	123 (82.6)	6 (85.7)	129 (82.7)
	Other	26 (17.4)	1 (14.3)	27 (17.3)
Blood transfusion history	Yes	10 (6.7)	0	10 (6.4)
	No	139 (93.3)	7 (100.0)	146 (93.6)
HIV	Positive	2 (1.3)	0	2 (1.3)
	Negative	147 (98.7)	7 (100.0)	154 (98.7)
Under five children in the household	None	87 (58.4 %)	3(42.9)	90 (57.7)
	1–2	59 (39.6)	3 (42.9)	62 (39.7)
	3+	3 (2.0)	1 (14.3)	4 (2.6)
History of abortion	Yes	43 (28.9)	2 (28.6)	46 (29.5)
	No	106 (71.1)	5 (71.4)	110 (70.5)
Parity	+1	62 (41.6)	2 (28.6)	64 (41.0)
	2–4	80 (53.7)	5 (71.4)	85 (54.5)
	5+	7 (4.7)	0	7 (4.5)

### Congenital and maternal cytomegalovirus infection

Overall, zero *rubella* and 2/156 [1.3 %; 95 % CI: 0.0–3.8] recent *cytomegalovirus* (CMV-IgM) infection was found among newborns. Out of the total 156 mothers 149 [95.5 %; 95 % CI: 92.3–98.7) had positive result for anti-CMV-IgG antibodies and 8 (5.5 %; 95 % CI: 1.3–9.0) were positive for CMV-IgM. Seven (4.5 %) mothers negative for CMV. These were categorized into four types of re-sponses. The first category was CMV-IgM positive newborns, most likely primary infection. The second category had previous exposure to CMV [IgG (+) plus IgM (−)]. This constituted 90.4 % of the women. The third group was those with active (primary/latent) infection [IgG (+) plus IgM (+)] and this consisted of 5.1 % mothers. The last category of women was those who had sero-negative [IgG (−) plus IgM (−)] test result (Table 2).

**Table 2:** Seroprevalence of CMV-specific IgG and IgM antibodies among newborn and their mother (n = 312) in St.Paul’s Hospital millennium Medical College, Addis Ababa, 2015

Immune response	Number %	Interpretation
IgM(+) newborn	2 (1.3)	Recent infection
IgG(+) IgM(−) mother	141 (90.4)	Previous exposure
IgG(+) IgM(+) mother	8 (5.1)	Active (primary/latent) infection
IgG(−) IgM(−) mother	7 (4.5)	Susceptible for primary infection

#### Risk factors

For analysis between risk factors and congenital cytomegalovirus infection, regression was not done due to less number of positive newborns.

Whereas, cytomegalovirus recent infection was significantly isolated from mothers with history of transfusion (25.0 %, OR 0.09, 95 % CI 0.0–0.3, P = 0.006) than have not been transfused. Mothers who had no transfusion history were 91 % odds less likely to acquire cytomegalovirus infection. History of abortion was also one of an independent risk factor (OR 0.02, 95 % CI 0.0–0.6, P = 0.023), in which, mothers with history of abortion were 98 % odds less likely to acquire cytomegalovirus infection. HIV positive mothers were 5.0 times having odds of Cytomegalovirus infection than sero-negative mothers (OR 5.0, 95 % CI 1.5–15.8, P = 0.034). Furthermore, Cytomegalovirus infection was significantly more prevalent in mothers who are in multi parity stage than null parity (OR 0.08, 95 % CI 0.01–0.7, P = 0.022) (Table 3).

**Table 3:** Association of maternal CMV—IgM with Obstetrical, socio-demographical and clinical characteristic of the mother (n = 156) in SPHMMC, Addis Ababa, Ethiopia 2015

Characteristics	CMV-IgM status		COR (95 % CI)	P value	AOR (95 % CI)	P value
	Positive n (%)	Negative n (%)				
Age						
+25	2 (25.0)	74 (50.0)	14.8 (1.7–128)	0.014	1.8 (0.1–32.0)	0.81
26–35	4 (50.0)	69 (46.6)	6.9 (1.01–47.0)	0.049	1.7 (0.13–23.9)	0.83
36+	2 (25.0)	5 (3.4)	1		1	
Income						
+1500	2 (25.0)	79 (53.4)	2.1 (0.2–24.1)	0.56	1.36 (0.1–18.1)	0.82
1500–2500	5 (62.5)	50 (33.8)	0.5 (0.1–4.8)	0.57	0.5 (0.05–5.2)	0.57
>2500	1 (12.5)	19 (12.8)	1		1	
Educational status						
Illiterate	3 (37.5)	31 (20.9)	2.9 (0.4–21)	0.30	0.8 (0.034–21.2)	0.90
Primary	1 (12.5)	40 (27.0)	11.4 (0.9–143)	0.06	8.7 (0.3–247.5)	0.20
Secondary	3 (37.5)	69 (46.6)	10 (1.2-82.3)	0.03	15.7 (0.5–485.0)	0.12
Higher education	1 (12.5)	8 (5.4)	1		1	
Occupation						
Housewife	6 (75.0)	123 (83.1)	1		1	
Other	2 (25.0)	25 (16.9)	0.6 (0.12–3.2)	0.60	0.6 (0.12–3.2)	0.60
Blood transfusion history						
Yes	2 (25.0)	8 (5.4)	5.8 (1.02–33.6)	0.048	0.09 (0.0–0.3)	0.006
No	6 (75.0)	140 (94.6)	1		1	
HIV-sero status						
Positive	1 (12.5)	1 (0.7)	0.048 (0.003–0.8)	0.038	5.0 (1.5–15.8)	0.034
Negative	7 (87.5)	147 (99.3)	1		1	
Under five children in the household						
None	4 (50.0 %)	86 (58.1)	1			
1–2	3 (37.5)	59 (39.9)	0.92 (0.2–4.2)	0.91		
3+	1 (12.5)	3 (2.0)	0.14 (0.01–1.6)	0.12		
History of abortion						
Yes	6 (75.0)	39 (26.4)	0.12 (0.02–0.6)	0.012	0.02 (0.0–0.6)	0.023
No	2 (25.0)	109 (73.6)	1		1	
Pregnancy frequency						
+1	1 (12.5)	63 (42.6)	1		1	
2–4	5 (62.5)	80 (54.0)	0.6 (0.12–3.7)	0.63	0.6 (0.12–3.7)	0.63
5+	2 (25.0)	5 (3.4)	0.08 (0.01–0.7)	0.022	0.08 (0.01–0.7)	0.022
Hearing loss						
Yes	1 (12.5)	1 (0.7)	0.05 (0.003–0.8)	0.038	0.01 (0.0–1.9)	0.08
No	7 (87.5)	147 (99.3)	1		1	

*COR* Crude Odds Ratio, *AOR* Adjusted Odds Ratio, *CI* Confidence Interval

Cytomegalovirus infection among the mothers: 48.7 % were between age groups of ≤25, 46.8 % were between 26-30, and 4.5 % were above 32.The infection prevalence among age groups ≤30, and ≥31 were 85.2 % (OR 2.3, 95 % CI 0.03–2.3), and 14.8 % respectively. However, based on the logistic regression analysis no statically significant variables were found. There was no significant association detected between CMV sero-prevalence and maternal educational, and occupation status (Table 4).

**Table 4:** Association of Anti-CMV antibodies with Obstetrical, socio-demographical and clinical characteristic of the mother (n = 156) in SPHMMC, Addis Ababa, Ethiopia 2015

Characteristics	CMV-IgG status		COR (95 %CI)	P value	AOR (95 % CI)	P value
	Positive n (%)	Negative n (%)				
Age						
+30	127 (85.2)	5 (71.4)	2.3 (0.42–12.6)	0.33	0.3 (0.03–2.3)	0.22
31+	22 (14.8)	2 (28.6)	1			
Income						
+1500	79 (53.0)	2 (28.6)	0.5 (0.04–5.6)	0.56	0.6 (0.1–7.5)	0.68
1500–2500	51 (34.2)	4 (57.1)	1.5 (0.16–14.2)	0.73	1.6 (0.16-16.5)	0.68
>2500	19 (12.8)	1 (14.3)	1		1	
Educational status						
Less than 8th grade	73 (49.0)	2 (28.6)	0.42(0.1–2.2)	0.30	0.4 (0.1–2.0)	0.25
More than 9th grade	76 (51.0)	5 (71.4)				
Occupation						
Housewife	123 (82.6)	6 (85.7)	1.3 (0.15–10.9)	0.83	1.8 (0.2–16.7)	0.61
Other	26 (17.4)	1 (14.3)	1			
Blood transfusion history						
Yes	10 (6.7)	0	NA			
No	139 (93.3)	7 (100.0)				
HIV						
Positive	2 (1.3)	0	NA			
Negative	147 (98.7)	7 (100.0)				
Under five children in the household						
None	87 (58.4 %)	3(42.9)	1			
1–2	59 (39.6)	3 (42.9)	1.5 (0.28–7.5)	0.64		
3+	3 (2.0)	1 (14.3)	9.7 (0.76–122.4)	0.08		
History of abortion						
Yes	43 (28.9)	2 (28.6)	0.9 (0.2–5.3)	0.99	0.8 (0.12–4.8)	0.78
No	106 (71.1)	5 (71.4)	1		1	
Pregnancy frequency						
+2	104 (41.6)	5 (71.4)	1		1	
3+	45 (53.7)	2 (28.6)	1.1 (0.2–5.8)	0.93	2.2 (0.24–20.3)	0.47

*COR* Crude Odds Ratio, *AOR* Adjusted Odds Ratio, *CI* Confidence Interval

## Discussion

The seroprevalence of CMV varies according to studies conducted in different parts of the world. But, it has been reported that 0.2 to 2 % of live birth have congenital CMV infection, considering CMV as the leading cause of congenital infections worldwide [13–15]. The fetus is at risk of acquiring CMV infection either through intrauterine or during delivery. Intrauterine transmission of CMV infection may occur following either primary or recurrent infection [16, 17]. Involvement of central nervous system (CNS), including late central nervous system sequelae, primarily sensory neuronal deafness is the most important clinical manifestation in 10–20 % of such CMV congenital infected infants [18]. Screening of mothers for CMV and early diagnosis play an important role to minimize CMV congenital infection and its serious consequences. However, to our knowledge this is the first data in Ethiopia concerning epidemiology of congenital CMV and *rubella* among newborns.

The present study showed that *Cytomegalovirus* specific IgM was detected in 1.3 % of the newborns in their umbilical cord. CMV-IgG antibodies evaluation in umbilical cord blood was not performed because of the absence of a discriminative test to identify congenitally infected newborns. This might be due to the high prevalence of CMV seropositivity in developing countries [19–21], and the possibility of IgG antibodies transmission from mothers to fetuses [22].

Our finding was comparable with other studies; 1.1 % in Brazil [23], 2 % in Havana, Cuba [24], 2 % in Iran [25]. However, it was in contrast to other findings; 0.1 % in Turkey [26], 0.9 % in Thailand [27], 0.9 % in Korea [28], 17.8 % in Egypt [29], 21.6 % in India [30], 18.7 % in Delhi [31], 4.8 % in Finland [32]. A higher frequency of CMV IgM detection (30 %) is normally found in newborns of mothers with poor obstetrical history or abnormalities during pregnancy [33]. Our findings differ from this observation, because the study participants were among healthy/asymptomatic newborns.

The relationship between maternal and fetal/neonatal infection has been mentioned that pregnant patients with active CMV infection were at risk of having congenitally infected children when compared with those in whom viral activity was not detected during pregnancy [24].

As the IgM does not cross the placental barrier, the IgM obtained in cord serum samples could originated from an active CMV infection occurring in babies during pregnancy or delivery. It is generally understood that pregnant women infected with CMV can transmit this infection during pregnancy, and risk is higher for a mother with primary infection (5–15 %) than for one with non-primary infection (<2 %) [34]. However this relationship could not be assessed in our study, since we could not differentiate women by type of infection.

Although, IgM assay is still considered as a reasonable tool for congenital CMV infection diagnosis [35], it was reported that only 45–80 % of babies congenitally infected with CMV could be recognized by detection of IgM [36]. Thus, looking at polymerase chain reaction usage for accurate and effective diagnosis of this pathogen is unquestionable.

In many developing countries, the burden of CRS is under-estimated [37]. Also Ethiopia lacks information regarding the burden of CRS, thus intensive types of research is important for the decision to introduce rubella-containing vaccine in the national immunization program. In this study, *Rubella* specific IgM was not detected from cord blood samples. This is in contrast with another study where it was present in 2 % of cases in Sudan [38]. This might be because our research was conducted among healthy/asymptomatic newborns, whereas the other study was on symptomatic children.

In this study the maternal prevalence of anti-CMV IgG was 95.5 %, which is comparable with other research findings in a similar setting [39] and different studies in developing countries such as; 77.3 % in Kenya [19], 97.5 % in Sudan [40], 96 % in Egypt [21], 92 % in Nigeria [41], and 87 % in Gambia [42].

Cytomegalovirus specific IgM has been detected from eight (5.1 %) mothers. This is in agreement with studies in other region; 4 % in Nigeria [41], 8.1 % in Kenya [19], 6 % in Sudan [40]. However, our finding is in contrast with other studies in similar setting [39] and 2.5 % in Iran [43], 1.7 % in Korea [44]. This might be because of high numbers of immune compromised participants and toddlers, socio-economic and geographical distribution difference. HIV-infected women are often CMV seropositive and experience more frequent CMV recurrences with progressive immune impairment [45]. The risk for infant mortality is increased in HIV-CMV-coinfected infants, and there is accelerated progression of CNS disease in survivors, especially developmental delay and worsening motor deficits [7, 46].

Multiple risk factors were identified between CMV—IgM positivity rate and maternal characteristics. Cytomegalovirus—IgM was significantly detected from mothers with history of transfusion, history of abortion, multi parity and HIV sero-positive. Similarly, those risk factors were observed in different studies [19, 40, 45]. In the current study age and socio-economic status were not significantly associated with CMV sero-positivity, while different studies suggest individuals with low income and elderly women were at higher risk of CMV-infection [47].

The limitations of our study include (i) the lack of long-term follow up data for infected infants; (ii) study design precluded inclusion of molecular technique and neonatal urine culture as a gold standard method; and (iii) the timing of maternal CMV seroconversion couldn’t be assessed by early pregnancy serology.

## Conclusion

In general, although this study showed low congenital Cytomegalovirus infection among newborns, there is high seroprevalence of CMV infection among mothers at our center; and is likely to be a reflection of the overall high prevalence among adult Ethiopians. These data might therefore; help to create awareness for clinicians in Ethiopia, and CMV associated complications should be taken into consideration during management of congenital related viral diseases. Future studies, including large scale surveillance throughout Ethiopia might be needed before national screening and universal prevention measures should be considered.

Although *Rubella* was not detected in this study, there is high number of individuals with congenital *Rubella* syndrome (CRS). Therefore, a large scale study should be conducted to recommend introduction of national *Rubella* immunization.

## Additional file

**Additional file 1.** Questioners.
