# Supplementary material for: Maternal and Congenital cytomegalovirus infection and zero rubella IgM prevalence in newborns in St.Paul’s Hospital Millennium Medical College
Source: BMC Res Notes. 2016 Oct 21;9:476. doi: 10.1186/s13104-016-2274-1 (PMC5073938; doi:10.1186/s13104-016-2274-1)
Supplement: Supplementary file 1 — Additional file 1. Questioners. [file 13104_2016_2274_MOESM1_ESM.doc]

# Questioners

St.Paul’s Hospital Millennium Medical College questioner form to assess the demographic characteristics and risk factor of cytomegalovirus infection among infants in St.paul’s Hospital Millennium Medical College, Ethiopia.

Medical Record No .............................Date of admission (in patient)

Address..................................................City, District....................

| S. No | Questions | Alternatives |
| --- | --- | --- |
| 1. **Socio-demographic characteristics of the Mother** | | |
| 1.1 | Age | ….........years |
| 1.2 | Sex | 1. 1. Male 2. Female |
| 1.3 | How money times did you pregnant? | 1. ------------------------------------------- |
| 1.4 | Parity | 1. ------------------------------------------------ |
| 1.5 | Marital Status | 1. Married 2. Not Married |
| 1.6 | Educational status | 1. Illiterate 2. Primary 3. Intermediate 4. Secondary 5. Diploma and above |
| 1.7 | Occupation | 1. House wife 2. Government and Non-government |
| 1.8 | Number of under five children in the house hold | 1. ---------------------------- |
| 1.9 | Monthly income in birr | 1. …………………… |
| 1.10 | Blood transfusion History | 1. 1. Yes 2. No |
| 1.11 | History of Abortion | 1. 1. Yes 2. No |
| 1.12 | HIV Sero status | 1. 1. Negative 2. Positive 3. Unknown |
| 1.13 | Do you have a handy caped child/hearing loss? | 1. 1. Yes 2. No |
| 1.14 | HBsAg-sero status | 1. 1. Positive 2. Negative 3. Undetermined |
| 1. **2. Clinical Informations of infants** | | |
|  |  |  |
| 2.1 | Wight | ----------------------------- |
| 2.2 | Height | 1. ----------------------------- |
| 2.3 | Head circumferences | 1. ----------------------- |
|  | | |

Interviewer name ….................................................. Date …...../…...../…...........

Signature …........................

**Thank you very much!!!**
